# Supplementary material for: The Nutraceutical Properties of Sumac (Rhus coriaria L.) against Gastritis: Antibacterial and Anti-Inflammatory Activities in Gastric Epithelial Cells Infected with H. pylori
Source: Nutrients. 2022 Apr 22;14(9):1757. doi: 10.3390/nu14091757 (PMC9104352; doi:10.3390/nu14091757)
Supplement: Supplementary file 1 [file nutrients-14-01757-s001.zip › nutrients-1702225-supplementary.pdf]

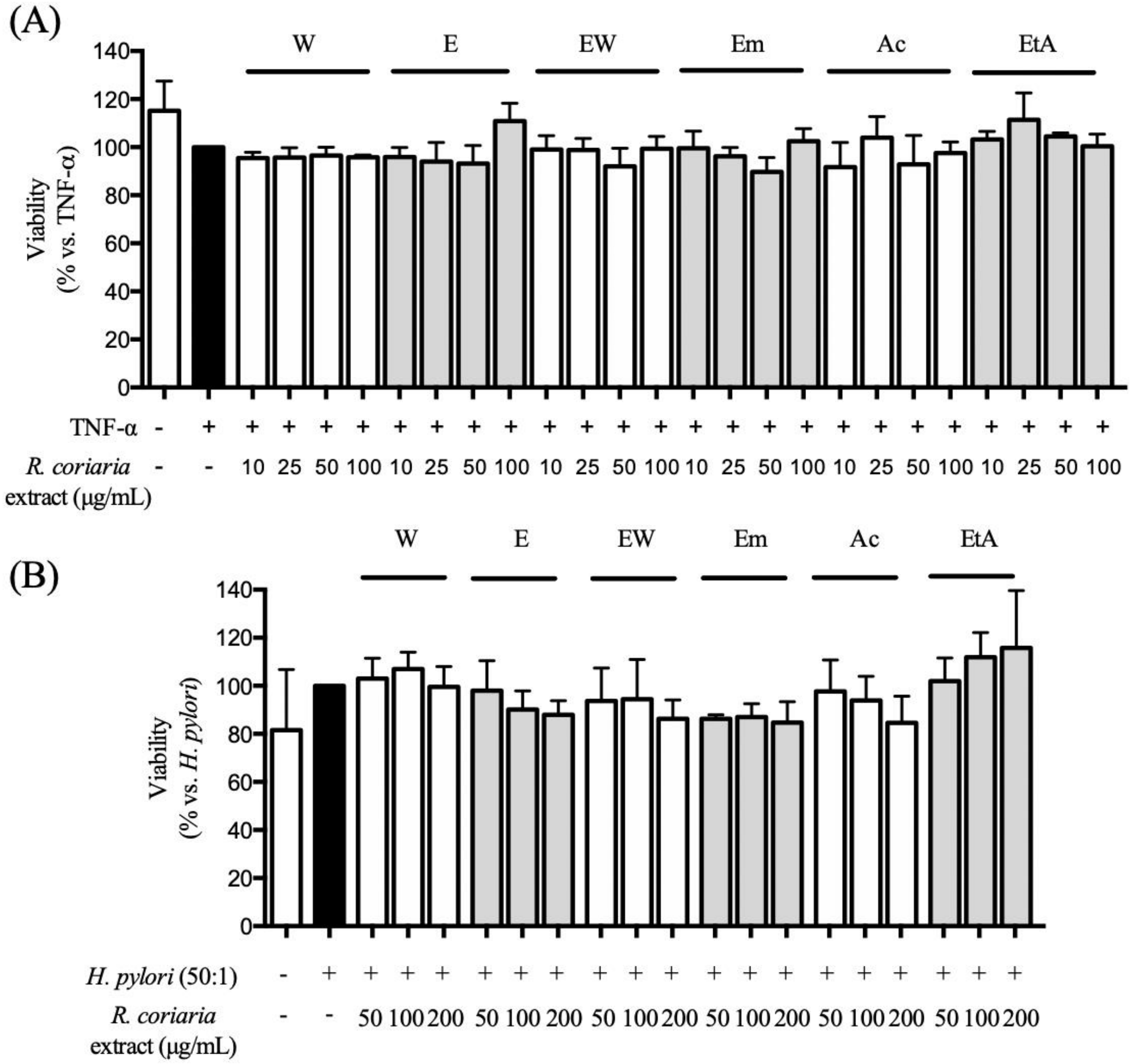

Figure S1. Evaluation of cellular viability of *Rhus coriaria* extracts. MTT assay was performed on *Rhus coriaria* L. extracts at concentrations ranging between 10 and 200 μg/mL. All the extracts did not show any toxicity in both GES-1 cells stimulated with TNF-α (**A**) and GES-1/*H. pylori* co-culture (**B**). MTT, 3-(4,5-dimethylthiazol-2-yl)-2-5-diphenyltetrazolium bromide; TNF-*α*, tumor necrosis factor alpha; W, Water; E, Ethanol; EW, Ethanol-water; Em, Macerated ethanol; Ac, Acetone; AtA, Ethylacetate; *R. coriaria*, *Rhus coriaria*; *H. pylori*, *Helicobacter pylori*; GES-1, gastric epithelial cells; − and +, absence or presence of the respective conditions.

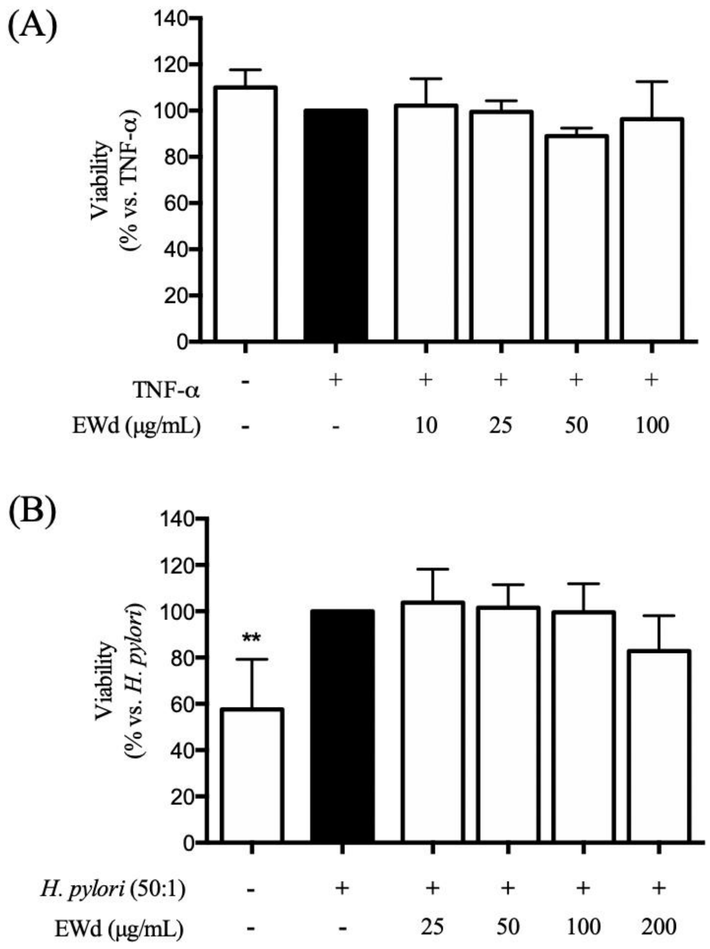

Figure S2. Evaluation of cellular viability of *Rhus coriaria* EWd extract. MTT assay was performed on *Rhus coriaria* L. ethanol-water digested (EWd) extract between 10 and 200 μg/mL. The extract did not show any toxicity in both GES-1 cells stimulated with TNF-α (**A**) and GES-1/*H. pylori* co-culture (**B**). EWd, EW extract subjected to in vitro simulated gastric digestion.
